# Toward Understanding the Molecular Recognition of Fungal Chitin and Activation of the Plant Defense Mechanism in Horticultural Crops

**DOI:** 10.3390/molecules26216513

**Published:** 2021-10-28

**Authors:** Yaima Henry García, Orlando Reyes Zamora, Rosalba Troncoso-Rojas, Martín Ernesto Tiznado-Hernández, María Elena Báez-Flores, Elizabeth Carvajal-Millan, Agustín Rascón-Chu

**Affiliations:** 1 Coordinación de Tecnología en Alimentos de Origen Vegetal, Centro de Investigación en Alimentación y Desarrollo, A.C., Carretera Gustavo Enrique Astiazarán Rosas No. 46, Col. La Victoria, Hermosillo C.P. 83304, Mexico; yaima.henry@estudiantes.ciad.mx (Y.H.G.); orlando.reyes@estudiantes.ciad.mx (O.R.Z.); tiznado@ciad.mx (M.E.T.-H.); arascon@ciad.mx (A.R.-C.); 2 Facultad de Ciencias Químico Biológicas, Universidad Autónoma de Sinaloa. Calle de las Américas y Josefa Ortiz de Domínguez, Culiacán C.P. 80013, Mexico; elenabf@uas.edu.mx; 3 Coordinación de Tecnología en Alimentos de Origen Animal, Centro de Investigación en Alimentación y Desarrollo, A.C., Carretera Gustavo Enrique Astiazarán Rosas No. 46, Col. La Victoria, Hermosillo C.P. 83304, Mexico; ecarvajal@ciad.mx

**Keywords:** chitin oligosaccharides, chitin elicitor receptors, plant immunity, horticultural crops

## Abstract

Large volumes of fruit and vegetable production are lost during postharvest handling due to attacks by necrotrophic fungi. One of the promising alternatives proposed for the control of postharvest diseases is the induction of natural defense responses, which can be activated by recognizing molecules present in pathogens, such as chitin. Chitin is one of the most important components of the fungal cell wall and is recognized through plant membrane receptors. These receptors belong to the receptor-like kinase (RLK) family, which possesses a transmembrane domain and/or receptor-like protein (RLP) that requires binding to another RLK receptor to recognize chitin. In addition, these receptors have extracellular LysM motifs that participate in the perception of chitin oligosaccharides. These receptors have been widely studied in *Arabidopsis thaliana *(*A. thaliana*) and *Oryza sativa *(*O. sativa*); however, it is not clear how the molecular recognition and plant defense mechanisms of chitin oligosaccharides occur in other plant species or fruits. This review includes recent findings on the molecular recognition of chitin oligosaccharides and how they activate defense mechanisms in plants. In addition, we highlight some of the current advances in chitin perception in horticultural crops.

## 1. Introduction

One of the most important problems for the horticultural industry that has a negative impact on food security is the postharvest decay of fruits and vegetables [1]. Fruits and vegetables are highly susceptible to decay caused by necrotrophic fungi, such as *Alternaria alternata* (*A. alternata*), *Botrytis cinerea* (*B. cinerea*)*,* and *Colletotrichum gloeosporioides* (*C. gloeosporioides*), among others, even under low temperature storage conditions [2,3]. The primary means to control fungal diseases is the use of synthetic fungicides; however, their use has caused concern due to their possible negative effects on human health, potentially toxic residues, long degradation period, and the induction of resistant strains [4]. In this sense, there is a need to control postharvest diseases. A promising alternative is the induction of natural plant defense mechanisms, which are a complex network of biochemical and molecular events that can limit the penetration and invasion of pathogens in plant tissue, thereby preventing or decreasing disease development [5,6].

The plant defense mechanism is very complex and genetically controlled. These defense responses begin when molecules associated with pathogens (pathogen-associated molecular patterns (PAMPs) or microbe-associated molecular patterns (MAMPs)) are recognized by plant membrane receptors (PRRs) and activate signal transduction to the nucleus and subsequently modify the transcriptional activity [7]. Chitin is present in the fungal cell wall, and it has been shown to participate in the plant–pathogen recognition phenomena [8,9] and in the activation of innate plant defense mechanisms [10]. The presence of receptors in plants that recognize chitin and chitin oligosaccharides (ChOs) has been widely demonstrated [11,12,13,14].

Currently, two families of chitin receptors have been reported in plants, namely receptor-like kinases (RLKs) and receptor-like proteins (RLPs), both of which are fundamental components for detecting MAMPs or PAMPs [15]. These membrane receptors that recognize chitin have been widely studied in *O. sativa* (rice) and *A. thaliana* (*Arabidopsis*). Chitin elicitor binding protein receptors, such as CEBiP, were the first chitin RLPs found in rice. Further, they are characterized by extracellular lysine domains that interact with an RLK for chitin recognition. CEBiP contains two extracellular LysM motifs and one transmembrane domain; however, it does not possess intracellular domains to initiate signal transduction and hence requires additional proteins [16,17]. On the other hand, RLKs, such as chitin elicitor receptor kinase (CERK1), are one of the main chitin receptors found in *Arabidopsis*. They include extracellular Lys domains that are probably involved in the interaction with PAMPs, a transmembrane domain, and a cytoplasmic kinase domain with basic auto phosphorylation/myelin protein (MBP) kinase activity, which can initiate a signaling cascade within the cell [18,19]. In addition to chitin receptor proteins, some studies have reported that there are other receptor proteins that are members of the lysine domain receptor-like kinase (LYK) family in *Arabidopsis* and play a role in the interaction with PAMPs [20]. When ChOs are recognized by PRRs, the signaling pathways starts and a complex defense response is activated, including the transcription of defense genes [21].

The recognition of ChOs by plant receptors has been reported mainly in *Arabidopsis* and rice; conversely, there are scarce studies in horticultural crops. According to some authors [22,23], ChOs are multicomponent transmembrane complexes that are related but differ in detail between species. Therefore, it is not clear how the molecular recognition of ChOs and the activation of downstream signaling occur in horticultural crops. The main objective of this review is to present an overview of recent findings regarding the molecular recognition of chitin oligosaccharides and how they activate defense mechanisms in plants. In addition, we highlight some of the current advances on how this phenomenon of recognition occurs in horticultural crops.

## 2. Molecular Mechanism of Plant Response to Pathogens

Over the course of evolution, plants have developed different strategies to resist and protect themselves from pathogen attacks. The first defense barrier in plants is the physical or structural barrier (waxes, cuticle, trichomes, etc.) that prevents pathogens and predators from invading the plant cell [23]. When plants become infected, several changes in the cuticle take place, which causes the activation of defense responses [24]. Once pathogens overcome the first line of defense, the second line of defense is initiated based on the cellular innate immunity, which allows the plant to resist or block the pathogen [25]. Among the main components that participate in this immune system are the PRRs found in the plasma membrane of plant cells, which recognize highly conserved molecular patterns called microbial-associated molecular patterns (MAMPs) or pathogen-associated molecular patterns (PAMPs). These are products secreted by microorganisms or released from their cell walls by hydrolytic enzymes during plant–pathogen interactions. MAMPs include peptidoglycans from Gram-positive bacteria, lipopolysaccharides from Gram-negative bacteria, glucans, proteins, or flagellin [26], while chitin and its oligosaccharides are considered PAMPs [27]. The perception of these molecular patterns during infection by pathogens triggers defense reactions known as PAMP-triggered immunity (PTI). In this regard, chitin and its ChOs are considered general elicitor compounds capable of inducing a defense response in plant cells [28,29].

**Figure 1:**
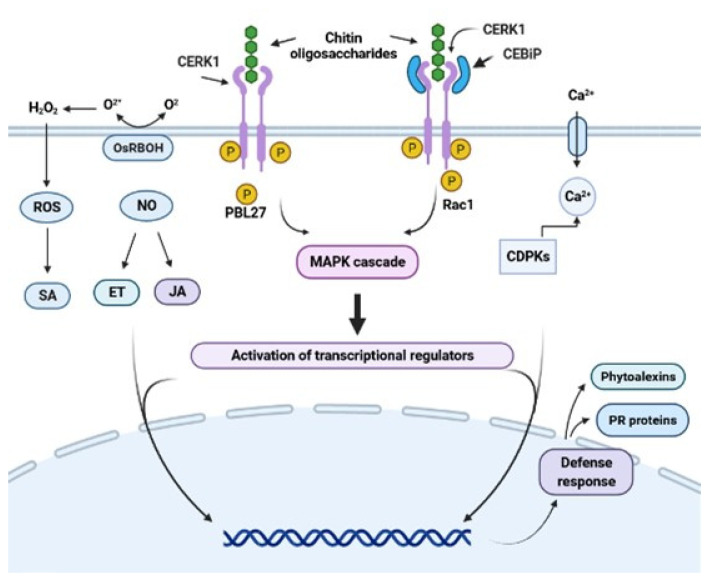
Representative scheme of chitin oligosaccharide perception by the chitin receptors RLK and RLP. Structural differences between the RLP (CEBiP) and RLK (CERK1) receptors are shown. Chitin oligosaccharides bind to CERK1 in *Arabidopsis*. This complex sends the signal to the intermediate protein, which will begin to phosphorylate and trigger the MAP kinase pathway, inducing the accumulation of SA, JA, ET, NO, and ROS and activating the defense responses. The CEBiP receptor in rice binds to CERK1, forming a heterodimer complex that will allow it to recognize chitin oligosaccharides and activate its defense mechanism in the same way. Figure created using BioRender (https://biorender.com/ accessed on 22 July 2021).

When MAMPs or PAMPs are recognized by PRRs, the defense responses are induced through the signaling cascade by mitogen-activated protein kinases (MAPKs) and calcium-dependent protein kinase (CDPK). The message induces the production of signaling hormones, such as salicylic acid (SA), jasmonic acid (JA), ethylene (ET), abscisic acid (ABA), auxins (AUX), cytokinin (CK), gibberellins (GB), and brassinosteroids (BR) [6,30,31]. Through these signaling pathways, a complex defense response is activated, including modifications to create structural defenses (random creation of bonds between cell wall polymers and lignification), the induction of reactive oxygen species (ROS), and nitric oxide (NO) (Figure 1). This activation has a role in transcriptional reprogramming and induction of high expression of genes related to early defense [32,33]. These genes encode pathogenesis-related (PR) proteins, such as glucanases, chitinases, peroxidases, and enzymes, such as phenylalanine ammonia-lyase, a key enzyme involved in the synthesis of phytoalexins [34,35], which play important roles in the defense against pathogens [6,36,37,38]. In this context, a successful plant defense mechanism should inhibit pathogen infection and protect the plant against future attacks.

## 3. Cellular Recognition of Fungal Molecules: Chitin

The fungal cell wall is made up of glycoproteins, glucans, and chitin, among other molecules. There is growing interest in chitin as this molecule has been shown to be essential in plant–pathogen recognition [9] and can induce activation of the innate plant defense mechanism [10]. Chitin molecules are composed of sugars linked by glycosidic bonds β (1→4), forming a linear chain of N-acetyl-2-amino-2-deoxy-D-glucose units, which are the second most abundant polymers in nature after cellulose [39,40]. Microscopically, it is in crystalline or semicrystalline form [41], making this polysaccharide a rigid and resistant material that acts as a very powerful barrier in the fungal cell wall and protects the organisms themselves [10]. Chitin is an insoluble polysaccharide when extracted by alkaline treatment and partially soluble when extracted by enzymatic treatment, from which other compounds of high biological significance are derived, such as chitosan and ChOs [42]. According to Buendia et al. [17] and Zhan et al. [43], ChOs are soluble depending on the pH and degree of polymerization (dp).

At the level of molecular recognition, some studies indicate the relationship of chitin and ChOs with the induction of plant defense mechanisms. Recent studies relate chitin as an enhancer of the biological control by *Rhodotorula mucilaginosa* (*R. mucilaginosa*) yeast against *Rhizopus* infection in peach crops. The addition of 0.5% of chitin reduced the postharvest disease of fruits by blue mold by up to half. In these fruits, differential expression of proteins was observed concerning the untreated fruits, including glucosidases, hydrolases, NADH dehydrogenases, and kinases with serine/threonine domains, which are decisive in the plant defense response against pathogen [44]. Similarly, the addition of chitin isolated from the yeast cell wall (*Saccharomyces cerevisiae*) in tomato fruit was correlated with increased expression levels of enzymes of oxidative metabolisms, such as the enzymes superoxide dismutase, peroxidase, and catalase, as well as enzymes inherent to the response to fungal infection, such as glucanase and chitinase [45].

## 4. Plant Membrane Receptors That Recognize Chitin and ChOs

Currently, two families of chitin receptors are reported in plants, namely RLKs and RLPs, which are fundamental components for detecting MAMPs or PAMPs [46,47]. These are related multicomponent transmembrane complexes, but they showed differences between species [22].

RLKs are a group of membrane proteins with an extracellular domain, including a sensor domain, a transmembrane region, and an intracellular region containing a Ser/Thr kinase domain with homology to protein kinases involved in signal transduction. Furthermore, they are divided into subfamilies according to the amount of lysine residues in their extracellular region [47]. On the other hand, RLP receptors lack an intracellular domain and, in some cases, are anchored to the membrane through a transmembrane domain of another protein or a glycosyl-phosphatidyl inositol group [17,29,48].

These membrane receptors of the RLK or RLP types have been widely studied and characterized in rice and *Arabidopsis*. In rice plants, chitin elicitor binding protein (CEBiP), which recognizes the N-acetyl groups of chitin by the lysine domains (LysM) of the receptor [21,49], was the first chitin receptor identified [16]. The molecular weight of this protein is estimated at 75 kDa, and it has 11 glycosylation sites through which N-acetyl glucosamine (GlcNAc) can bind. According to some authors, in rice plants, two receptors are involved in chitin-activated immunity [50]. LysM-RLP (OsCEBiP) binds to N-acetylated chitin fragments, initiating receptor homodimerization and further heterodimerization with OsCERK1, initiating PTI [51]. Additionally, the authors showed that in the OsCEBiP receptor, the central lysine motif is essential for recognizing ChOs with a dp of 8. CEBiP is dimerized with the OsCERK1 protein for binding to these oligosaccharides (Figure 1). In this sense, the chitin receptor system in rice requires both CEBiP and OsCERK1 for chitin sensing and signaling.

In *Arabidopsis*, a family of proteins related to chitin perception is found, and the CERK1 receptor is the most important within the RLK receptor family. LysM-RLK in *Arabidopsis* (AtCERK1) binds to N-acetylated chitin fragments with three LysM motifs and mediates the plant defenses induced by chitin through the formation of homodimers [12,18]. Important findings to understand the function of the CERK1 receptor in *Arabidopsis* were made by Petutsching et al. [13]. It was confirmed that in the absence of CERK1, no defense response was induced in *Arabidopsis* exposed to chitin and CERK1 showed the highest affinity for chitin. Additionally, the authors highlighted the importance of the lysine domains and the phosphorylation of CERK1 in the ectodomain for the recognition process of ChOs and the intracellular domain to initiate signal transduction.

According to some studies, the recognition of ChOs is more effective depending on their polymerization and acetylation degrees. For instance, in the cork tree *Hevea brasiliensis*, a linear relationship of 1:1 for (GlcNac)_5_ with the receptor was shown, and for (GlcNac)_8_, the ratio was 2:1 protein–oligosaccharide, indicating dimerization of receptors for the recognition of ChOs with higher dp [11]. (GlcNac)_6-8_ was recognized by membrane receptors in *Arabidopsis* [52]. In another study, (GlcNac)_5-9_ was recognized with great efficiency by the CERK1 receptor of *Arabidopsis* [12], while ChOs with a lower dp did not show affinity for the CERK1 receptor [13]. Later, Liu et al. [53] described the X-ray crystal structure of the AtCERK1 ectodomain and predicted the interaction of ChOs with the second LysM motif of the extracellular domain. These authors suggested that long chain chitin oligomers (dp ≥ 6) bind to LysM domains in two monomers, resulting in the homodimerization of AtCERK1. This dimerization was shown to activate the intracellular kinase domain [13,53]. However, there is a possibility that, as in rice, the active chitin receptor in *Arabidopsis* can be a complex of more than one protein [22].

In addition to chitin receptor proteins, members of the lysine domain receptor-like kinase (LYK) family have been reported in *Arabidopsis*. The plant LysM proteins mostly function as pattern recognition receptors (PRRs) that recognize chitin to induce the plant’s immunity [26]. Cao et al. [20] identified a LysM-RLK in *Arabidopsis* (AtLYK5) that binds to chitin with a higher affinity than AtCERK1. The authors proposed that AtLYK5 functions as the major chitin receptor, recruiting AtCERK1 to form a chitin-inducible receptor complex, which agrees with the results reported by other authors [54]. Erwig et al. [55] demonstrated that CERK1 is essential for recognizing ChOs in *Arabidopsis* and reported other receptor proteins involved in chitin perception. Specifically, LYK5 was shown to be required for the phosphorylation and dimerization processes of the CERK1 receptor. The authors concluded that CERK1, LYK4, and LYK5 were inducible proteins and that their presence in the cytoplasmic membrane was due to the presence of chitin and depended on a complex network of vesicular trafficking. Therefore, there are proteins that bind the intracellular domains of receptors and activate the signaling cascade, and kinase activity is required to activate immune responses [22,53]. For example, PBL27 is the downstream component of AtCERK1 after chitin perception, and it can be phosphorylated by AtCERK1 [56]. PBL19 and PBL27 phosphorylate MAPKKK to activate MAPK cascades, which in turn activates defense genes in the nucleus [57]. In summary, the receptors for these chitin patterns, such as CEBiP/CERK1, are activated by ligand binding and trigger various immune responses.

## 5. Recognition of Chitin and Its Oligosaccharides in Horticultural Crops

The chitin receptor complex and the relationship among the components have been mostly studied in rice and *Arabidopsis* model systems. However, few studies have been performed to investigate the presence of chitin receptors and the mode of action in defense activation in horticultural crops. For instance, Zhang et al. [58] made important contributions to understanding the biological function of a chitin receptor-binding protein in banana (*Musa acuminata*). The authors confirmed the presence of the MaLYK1 protein in the plasma membrane. This protein is a LysM domain chitin receptor that belongs to a subclade represented by AtCERK1 (AtLYK1) and OsCERK1 (OsLYK9). The expression of MaLYK1 was found to be higher in banana roots, while it was lower in yellow fruit. In addition, MaLYK1 expression was induced by *Fusarium oxysporum* f. sp. *cubense* race 4 (Foc4), and its function was essential not only in recognizing the fungus but also in recognizing the fungus in the symbiotic relationship established between plants and mycorrhizae. Besides, chitin treatment (GlcNAc)_8_ induced the expression of defense genes, such as phenylalanine ammonia-lyase, β-1,3-glucanase, and pathogenesis-related protein 1. Similar studies reported the activation of the MdCERK1 receptor in apple (*Malus domestica*) during *Rhizoctonia solani* infection, observing that MdCERK1 expression increased in roots and leaves. This protein is found in the plasma membrane and includes three lysine-rich domains in its extracellular zone, a transmembrane domain, and an intracellular domain with serine/threonine motifs [59]. Recently, another CERK gene, designated as *MdCERK1–2*, was identified in shoot barks of apples. It encodes a protein with high similarity with the previously reported MdCERK1 and AtCERK1. Results revealed that *MdCERK1–2* expression in apple was induced by *Botryosphaeria dothidea* and *Glomerella cingulate*. Additionally, it was observed that the *MdCERK1–2* overexpression in transgenic *Nicotiana benthamiana* plants improved their resistance to *A. alternata* infection. These results suggest that *MdCERK1–2* is involved in apple defense responses against pathogenic fungi [60].

In tomato (*Solanum lycopersicum*), Bti9 interacts with the AvrPtoB protein from *Pseudomonas syringae* [61]. Bti9 is a protein kinase with 76% similarity with LysM CERK1 found in *Arabidopsis,* which belongs to the same clade as SlLYK11, SlLYK12, and SlLYK13. The authors suggested that these proteins are involved in the response to PAMPs that trigger the signaling cascade. Liao et al. [62] showed the relationship between homologous CERK1 receptors in tomato (SlLYK1, SlLYK12, and SlLYK13) and colonization of arbuscular mycorrhizae (AMs). The authors found that *SlLYK1* gene expression was upregulated after chitin oligosaccharide treatment, whereas *SlLYK12* was involved in AM symbiosis. Another interesting piece of data to elucidate the phenomena of chitin recognition showed that cell death was induced by overexpressing SlLYK13, suggesting that the chitin receptor genes have distinct functions in plants depending on the specific plant tissue or PAMPs applied. As well as in *Arabidopsis*, when ChOs are perceived in tomato, there are proteins that bind the intracellular domains of the chitin receptor and activate the signaling cascade, which includes kinase activity required for the activation of immune responses [22]. In tomato, the TPK1b protein is probably an ortholog of BIK1 in *Arabidopsis*, which is essential in the defense response of *Arabidopsis* against *B. cinerea*. The susceptibility to *B. cinerea* was increased when TPK1b was inhibited by RNAi [63]. Another study found that in the defense response mediated by systemin, TPK1b was phosphorylated by the ortholog of RLK1 (PORK1). In the same study, when PORK1 was silenced, the levels of TPK1b phosphorylation decreased to the same extent, which affected the defense response through this pathway [64]. These studies further support the essential role of TPK1b in the transduction of the RLK receptor signal in various plant defense responses.

In summary, it was found that *Arabidopsis thaliana* and *Oryza sativa* have a group of receptors with high affinity for ChOs and that the interactions for this recognition is fairly well understood. In the same way, one of these receptors (RLK type) was described in certain horticultural crops in which the structural patterns are preserved. Additionally, the role of ChOs in the activation of the defense mechanism was observed in horticultural crops.

## 6. Concluding Remarks and Future Perspectives

Great advances have been made to elucidate the molecular recognition of chitin oligosaccharides by plant membrane receptors and the functional receptor complex based on studies performed in the model plants *Arabidopsis thaliana* and *Oryza sativa*. According to the few studies reported in horticultural crops, it is possible to suggest that the molecular mechanism of chitin oligosaccharide perception occurs through RLK-type receptors with lysine domains, such as the CERK1 receptor reported in *Arabidopsis*. This suggests that the chitin receptors reported in model plants such as *Arabidopsis* and rice could be conserved in other plant species. Nevertheless, details of the receptor complex formation induced by chitin oligosaccharides and the activation of signaling and defense responses within the cell are not clear. Therefore, future studies are required to fully elucidate the molecular mechanism of the receptor role in the response to pathogen attack.

Understanding the molecular mechanism of pathogen recognition and the activation of the defense mechanism would help with the problem of horticultural postharvest losses and food supply by providing safe and effective alternatives for the development of disease-resistant crops and/or food preservation without using synthetic chemical compounds.

## Data Availability

Not Applicable.
